# Modified Fletcher's 3-channel brachytherapy system with vaginal line source loading versus uterine tandem and vaginal cylinder system in Stage IIIA cervical cancer

**DOI:** 10.2349/biij.2.1.e15

**Published:** 2006-01-01

**Authors:** JSH Low, KB Ng

**Affiliations:** 1 Gynec-Oncology Unit, Department of Radiation Oncology, National Cancer Centre, Singapore; 2 Medical Physics Unit, Department of Radiation Oncology, National Cancer Centre, Singapore

## Abstract

**Purpose:**

The uterine tandem with open-ended vaginal cylinder is the most commonly used brachytherapy system for Federation Internationale de Gynecologie et d'Obstetrique (FIGO) Stage IIIA cervix cancer at the National Cancer Centre, Singapore. Without the 3-channel ovoid system, the dose to the parametrium is often compromised. In this study, a vaginal cylinder that could potentially be incorporated with the 3-channel system was developed, hence addressing the problem of treating both the vaginal disease extension and the parametrium.

**Methods and materials:**

A hollow cylinder of 3 cm in diameter was incorporated with the Fletcher's 3-channel tandem and ovoid system. Treatment plans were generated with the single tandem line source with a vaginal cylinder applicator and the modified Fletcher's system using the Abacus version 3 brachytherapy treatment planning software. A nominal dose of 5 Gy was prescribed to point H for both plans. The perpendicular distance of the 5 Gy isodose line from the uterine tandem plane at the centre of the ovoid and the vaginal cylinder plane 1 cm below the os guard were then compared.

**Results:**

The 5 Gy isodose line was 1.7 cm from the uterine tandem source at the location lateral through the centre of the ovoids on the plan with the uterine tandem and vaginal cylinder system as compared to a distance of 3.3 cm using the modified 3-channel Fletcher system. The 5 Gy isodose line was 2 cm lateral to the central source at the vaginal cylinder plane 1 cm below the os guard on the uterine tandem and vaginal cylinder system as compared to a distance of 2.5 cm on the Modified-Fletcher system. This corresponds to an increase of 1.6 cm and 0.5 cm depth of treated parametrium on the uterine tandem plane and vaginal cylinder plane respectively with the modified Fletcher's applicator as compared with the single line source cylinder system.

**Conclusion:**

As compared with the single uterine tandem and open-ended vaginal cylinder system, an addition of 1.6 cm of the parametrium was covered within the 5Gy isodose on the uterine tandem plane and 0.5 cm on the vaginal cylinder plane with the modified Fletcher's applicator. A feasibility study was started to address the ease of insertion of this modified Fletcher system into patients.

## INTRODUCTION

**Figure 1:**
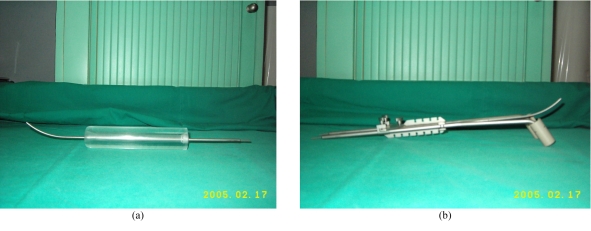
(a) Uterine tandem with vaginal cylinder system. (b) Fletcher's 3-channel system.

FIGO IIIA cervix cancer with disease extension down to lower third of vagina, without extension onto the pelvic sidewall or hydronephrosis is uncommon and constitutes approximately 2% to 3% of all cervical cancers [1,2]. The current standard of care consists of external beam radiotherapy to the whole pelvis followed by brachytherapy (intracavitary or implants) with concurrent single agent cisplatin chemotherapy. The uterine tandem with vaginal cylinder is the most commonly used brachytherapy system as it deals with the disease extension onto the lower third of the vagina (Figure 1a). The Fletcher's 3-channel brachytherapy system with ovoids is not used, as the disease in the lower vagina cannot be addressed (Figure 1b). Parametrial disease extension and discontinuous involvement of the vagina are poor prognostic features in stage III disease [2]. Without the 3-channel ovoid system, the radiation dose to the parametrium may be compromised. In this study, a vaginal cylinder that could potentially be incorporated with the 3-channel system was developed. This report is a pilot dosimetric study to assess the applicability of this new vaginal applicator.

## METHODS AND MATERIALS

**Figure 2:**
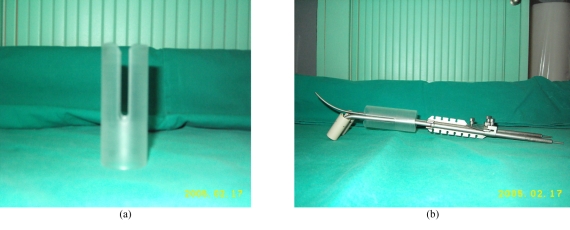
(a) Hollow Perspex cylinder fashioned in our workshop (rectangular slits to accommodate the ovoids). (b) Perspex cylinder incorporated into the 3-channel Fletcher's system.

A hollow Perspex cylinder of 3 cm in diameter was fashioned. The ends of the cylinder were smoothened out to avoid injury to the vagina mucosa (Figure 2a). Two open ended rectangular shaped slits were fashioned at the end of the cylinder to accommodate the ovoids. The cylinder was then incorporated with the Fletcher's tandem and ovoids systems (Figure 2b). The separation between the ovoids was set at 3.5 cm, the usual distance in the 3-channel brachytherapy treatment. The 3-channel brachytherapy with vaginal applicator was placed in air and orthogonal films taken in the simulator for dosimetric calculation. Similarly, the open-ended vaginal cylinder with a single uterine tandem applicator was placed in air for orthogonal films. The Abacus version 3 brachytherapy treatment planning system for Gammamed brachytherapy machine (Isotopen-Technik Dr Sauerwein GmBH) was used for dose calculation. A nominal dose of 5 Gy was prescribed to point H for both plans [3]. The separation between the sources was 5 mm apart. The HDR system was used and the basis of source loading was according to variable dwelt time generated by the Abacus 3 planning system (Isotopen-Technik Dr Sauerwein GmBH). The perpendicular distance of the 5 Gy isodose line from the uterine tandem plane at the centre of the ovoids and the vaginal cylinder plane 1 cm below the os guard were then compared to determine the amount of parametrial tissue covered within the 5 Gy volume between the two systems.

## RESULTS

**Figure 3:**
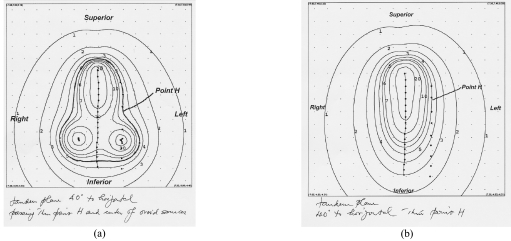
Distance of 5Gy isodose line from central source at Point H (uterine tandem plane 40° to horizontal). Scale: distance between adjacent points is 1 cm . (a) Flectcher's 3-channel system. (b) Uterine tandem with cylinder system.

**Figure 4:**
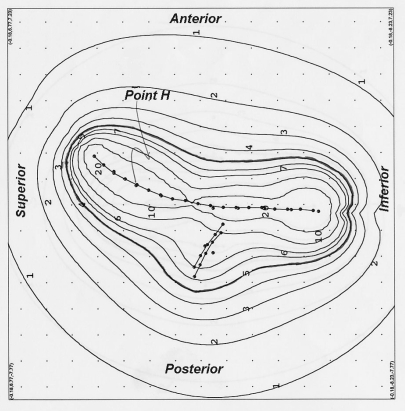
Sagittal plane of isodose distribution for 3-channel system with vaginal line source. Scale: distance between adjacent points is 1cm.

**Figure 5:**
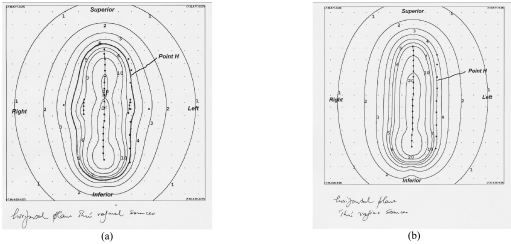
Distance of 5Gy isodose line from central source at vaginal cylinder plane. (a) Flectcher's 3-channel system. (b) Uterine tandem with cylinder system.

The distance of the 5 Gy isodose line was 1.7 cm from the uterine tandem source at the location lateral through the centre of the ovoids on the plan with the uterine tandem and vaginal cylinder system as compared with a distance of 3.3 cm using the modified 3-channel Fletcher system (Figure 3). Figure 4 shows the sagittal plane of the isodose distribution for the modified 3-channel system. The 5 Gy isodose line was 2 cm lateral to the central source at the vaginal cylinder plane 1 cm below the os guard on the uterine tandem and vaginal cylinder system as compared to a distance of 2.5 cm on the Modified-Fletcher system (Figure 5). This corresponds to an increase of 1.6 cm and 0.5 cm depth of treated parametrium on the uterine tandem plane and vaginal cylinder plane respectively with the modified Fletcher's applicator as compared with the single line source cylinder system. The results are summarised in Table 1.

**Table 1:** Distance of 5 Gy isodose line from central source.

**Uterine tandem plane at centre of ovoids (Figure 3)**	**Left (cm)**	**Right (cm)**	**Mean (cm)**
Fletcher's 3-channel	3.1	3.5	3.3
Uterine tandem with vaginal cylinder	1.7	1.7	1.7

**Vaginal cylinder plane 1 cm below os guard (Figure 4)**	**Left (cm)**	**Right (cm)**	**Mean (cm)**
Fletcher's 3-channel	2.5	2.5	2.5
Uterine tandem with vaginal cylinder	2.0	2.0	2.0

## DISCUSSION

3-channel uterine tandem with vaginal ovoids is the most commonly used brachytherapy system for cervical cancers. This treatment gives rise to the classical pear-shaped isodose distribution that adequately covers the disease in the cervix and parametrium. Study has shown that parametrial disease extension and discontinuous involvement of the vagina to be poor prognostic features in stage III disease [2]. Often the local disease failure in surgical treatment of cervix cancer is a lack of a clear margin in the parametrial sidewall. Radiotherapy has an advantage over surgery especially in more advanced stages of cervix cancer, as radiation is able to cover the parametrial tissue and pelvic sidewall adequately with external beam radiotherapy followed by a booster dose of brachytherapy. However, in Stage IIIA cancer with disease extension onto the vagina, single line source uterine tandem with open-ended vaginal cylinder is often used, as the Fletcher's 3-channel system cannot deal with the disease in the vagina. This is due to a lack of an apparatus to prevent the vaginal mucosa from collapsing onto the central tandem source. Although parametrial boost using external beam photons can be used, they do not have the advantage of rapid fall-off of doses and tissue sparing as compared with brachytherapy. There is also a risk of overlapping of the parametrial boost external beam with the brachytherapy treatment fields. A simple Perspex hollow cylinder was therefore fashioned in our workshop to be incorporated together with the 3-channel tandem-ovoid system. The purpose of the Perspex vaginal cylinder is to hold the vagina mucosa away from the tandem source, hence keeping the tandem source in the centre of the vagina. This will enable the loading of sources in the tandem down to the vagina. The cervical, parametrial and vagina disease can be targeted simultaneously with this modified 3-channel brachytherapy system.

Comparing the isodose plans between the 2 systems, there was a 1.6 cm (uterine tandem plane, Figure 3) and a 0.5 cm (vaginal cylinder plane, Figure 5) increase in the parametrial tissue covered within the 5 Gy isodose with this applicator as compared with the single line source uterine tandem with open-ended cylinder system.

## CONCLUSION

This simple dosimetric study showed that by adding the vaginal Perspex cylinder applicator on the 3-channel Fletcher's system, the conventional pear-shaped distribution of the isodoses is achieved along with the treatment of the disease extension onto the vagina at the same time.

This may be especially helpful in patients with Stage IIIA or more advanced disease with a combination of parametrial and vaginal involvement. A feasibility study was started to address the ease of insertion of this modified Fletcher system into patients with further modification as necessary before starting a perspective study on patients with Stage IIIA disease.
